# Prediction of functional recovery by cardiac magnetic resonance feature tracking imaging in first time ST-elevation myocardial infarction. Comparison to infarct size and transmurality by late gadolinium enhancement

**DOI:** 10.1186/1532-429X-17-S1-P87

**Published:** 2015-02-03

**Authors:** Sorin Giusca, Birgit Krautz, Yannick Sander, Lukas Rust, Christian Galuschky, Sebastian A Seitz, Evangelos Giannitsis, Sven Pleger, Philip Raake, Patrick Most, Grigorios Korosoglou, Hugo Katus, Sebastian Buss

**Affiliations:** Cardiology, University Hospital Heidelberg, Heidelberg, Germany; TomTec Imaging Systems, Munich, Germany

## Background

To investigate whether myocardial deformation imaging, assessed by a noninvasive post-processing feature tracking algorithm (FTI) on regular cardiac magnetic resonance (CMR) cine images, would allow objective quantification of regional and global left ventricular (LV) strain and provide the estimation of functional recovery in patients with first time ST-elevation myocardial infarction (STEMI).

## Methods

Cardiac magnetic resonance (CMR) imaging was performed in 74 consecutive patients (mean age: 57 ± 12yrs.) 2-4 days after successfully reperfused STEMI, using a 1.5T whole body MR scanner (Philips Achieva). Peak systolic circumferential and longitudinal strain were measured using the feature tracking algorithm applied to SSFP cine sequences and were compared to infarct size, determined by late gadolinium enhancement (LGE). Follow-up CMR at 6 months after STEMI was performed in order to assess follow-up ejection fraction, which deemed as the reference standard for the estimation of function.

## Results

Significant correlations were observed between global circumferential and longitudinal strain with infarct size by LGE (r=0.75, 95%CI=0.63-0.83 and r=0.45, 95%CI=0.24-0.61, respectively). During the follow-up period 53 of 74 (72%) patients exhibited preserved residual ejection fraction≥50%. A cut-off value of -19.3% for global circumferential strain identified patients with preserved ejection fraction ≥50% at follow-up with sensitivity of 76% and specificity of 85% (AUC=0.86, 95%CI=0.75-0.93, p<0.001), which was superior to that provided by longitudinal strain (ΔAUC=0.13, SE=0.05, zstatistic= 2.5, p=0.01), and non-inferior to that provided by LGE (ΔAUC=0.07, p=NS). Multivariate analysis showed that global circumferential strain and LGE exhibited independent value for the prediction of preserved LV-function, surpassing that provided by age, diabetes and baseline ejection-fraction (HR=1.4, 95%CI=1.0-1.9 and HR=1.4, 95%CI=1.1-1.7, respectively, p<0.05 for both).

## Conclusions

Estimation of circumferential strain by FTI provides objective assessment of infarct size without the need for contrast agent administration and estimation of functional recovery with non-inferior accuracy compared to that provided by LGE.

## Funding

N/A.

